# Florida manatees *Trichechus manatus latirostris* actively consume the sponge *Chondrilla caribensis*

**DOI:** 10.7717/peerj.8443

**Published:** 2020-01-22

**Authors:** William Fitt

**Affiliations:** Odum School of Ecology, University of Georgia, Athens, GA, USA

**Keywords:** Manatee, Predation, Sponges

## Abstract

The Florida manatee (*Trichechus manatus latirostris* Linnaeus 1758) actively selects and consumes the “chicken-liver” sponge *Chondrilla caribensis*. Manatees ate over 10% of *C. caribensis* on a sample dock, mostly from pylons that received no direct sunlight. Since manatees reportedly eat mostly seagrasses and algae, it was thought that the chlorophyll*-a* content of the symbiotic cyanobacteria in *C. caribensis* might be correlated to the amount eaten; however the correlation was not significant (*P* > 0.05)*. C. caribensis* has variable chemical defenses and round spherasters (spicules), but these do not appear to be effective deterrents to predation by manatees. This is the first direct evidence that manatees actively seek out and consume a sponge.

## Introduction

Florida manatees (*Trichechus manatus latirostris*) are generally considered to be strictly herbivorous mammals (e.g., seagrasses, algae, seeds, acorns; O’Shea, 1986; Guterres-Pazin, Rosas & Marmontel, 2012), only incidentally consuming invertebrates or fish (Powell, 1978). However, in spite of the fact that stomach and fecal analyses find mostly seagrasses and algae, there have been a few instances where manatees have been observed to eat invertebrates (e.g., Courbis & Worthy, 2003). Barnacles, tunicates, bivalves, gastropods, crustaceans, polychaetes, other small invertebrates and fish have been ingested by manatees and dugongs (Powell, 1978; Reeves, Tuboku-Metzger & Kapindi, 1988; Anderson, 1989; O’Shea et al., 1991; Preen, 1995; Courbis & Worthy, 2003; Guterres-Pazin, Rosas & Marmontel, 2012; Vélez-Juarbe, 2014). Spicules of sponges seen in necropsy samples obtained from manatee carcasses are generally considered incidentally ingested with seagrasses and algae (e.g., Allen et al., 2017). In addition, Florida manatees ingest undigestible debris while feeding, often killing the animal (Beck & Barros, 1991).

*Chondrilla caribensis* (= *C. nucula* “chicken liver sponge”), an encrusting sponge, has photosynthetic cyanobacteria and non-photosynthetic bacterial symbionts (Wilkinson & Vacelet, 1979; Hill et al., 2006), releases nitrate onto reef ecosystems (Corredor et al., 1988), and is an important competitor for space in tropical mangrove ecosystems in Florida, Bahamas, and the Caribbean (Hill, 1998). Presumably, symbiotic cyanobacteria conduct photosynthesis, fix nitrogen, and metabolize ammonia (oxidizing it to nitrate) that is excreted by the sponge (Wilkinson & Fay, 1979; Corredor et al., 1988). Sponges have variable concentrations of anti-predation compounds and structural defenses in their tissues (Jones, Blum & Pawlik, 2005). *C. caribensis* inhibits growth in chickens (Yacowitz & Zaccone, 1982) and is reported to be toxic to fish (Green, 1977), but tends to be eaten by reef fish more than most other sponges (Chanas & Pawlik, 1995). In fact, *C. caribensis* is the most frequently found sponge in the digestive tracts of fish (Randall & Hartman, 1968) and when transplanted from mangroves with few fish predators to the reef with lots of fish predators, it is completely consumed by fish (Swearingen & Pawlik, 1998). *C. caribensis* is also the main sponge eaten by hawksbill (Meylan, 1988; Pawlik, Loh & McMurray, 2018) and green (Bjorndal, 1990) turtles.

**Figure 1:**
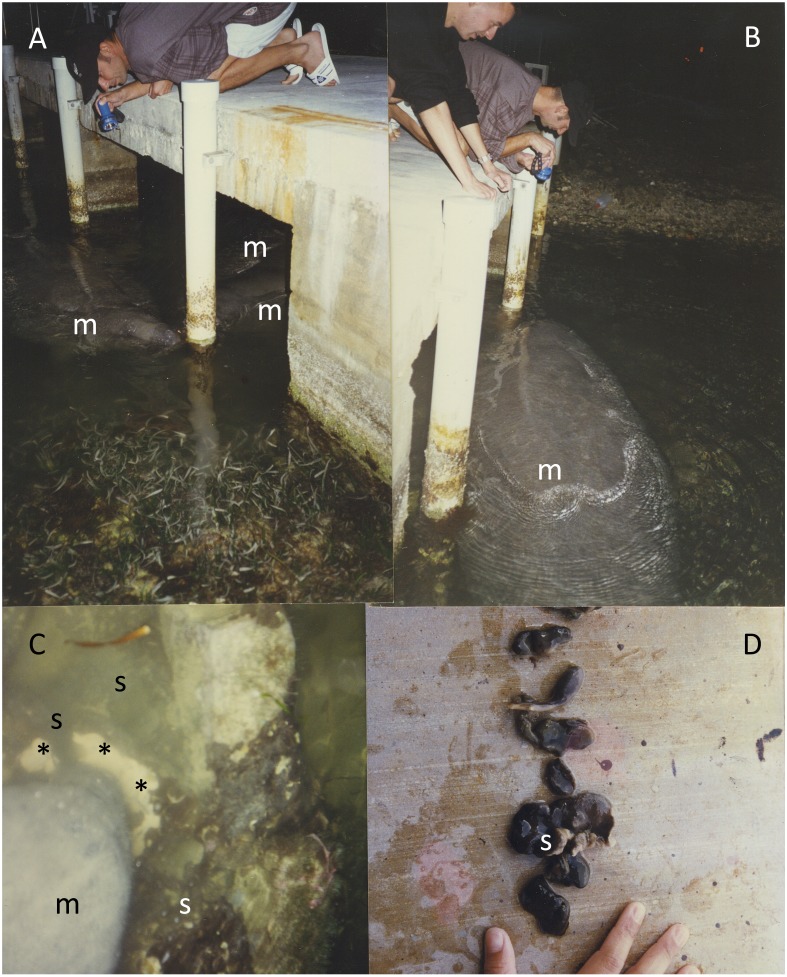
Manatees eating *C. caribensis* off pylons of a dock in Key Largo, Florida. Manatees eating *C. caribensis* off pylons of a dock in Key Largo, Florida. (A) Three manatees, (B) one manatee, (C) a manatee eating *C. caribensis* (m = manatee, * white is the sponge tissue, grey-brown is the symbiotic cyanobacteria in the outer layers of the sponge), (D) pieces of *C. caribensis*, showing its grey-brown external color that is due to the symbiotic cyanobacteria in the sponge.

*Chondrilla caribensis* has a low spicule content to organic matter ratio, which is thought to be less of a deterrent to certain predators (Randall & Hartman, 1968; Wulff, 2006). The siliceous spicules are round spherasters (ca. 28.8 um) with about 25 spines on each, plus minute secondary spines that are identified with scanning electron microscopy (Rützler, Duran & Piantoni, 2007). All of the sponges in the genera are rich in amino acids, protein (Yacowitz & Zaccone, 1982), and collagen (Rützler, Duran & Piantoni, 2007), possibly reasons why manatees and fish eat the sponge. Chlorophyll-*a* and phycobilin concentrations in bacterial symbionts living in *C. caribensis* determine the color variations of the sponge, which range from whitish or cream color (sponge) to dark grey-brown (sponge + symbionts) (Fig. 1). It is not known whether any of these factors are important in the nutrition of manatees.

Researchers, students and lay-people have observed manatees eating *C. caribensis* off docks, pylons and hard surfaces in the Florida Keys for at least twenty-five years (1992–2017). This paper describes intentional feeding on the sponge *C. caribensis* by the Florida manatee *T. manatus latirostris*. The following hypotheses were addressed: (H1) Manatees “select” the sponge *C. caribensis* from others, (H2): Manatees eat varying amounts (>10%) of *C. caribensis*, (H3): Manatees prefer eating *C. caribensis* in the shadiest area of the sample dock, (H4): Manatees eat *C. caribensis* containing more chlorophyll.

## Material and Methods

Observations of Florida manatees eating *C. caribensis* were made in all months of the year, for multiple years between 1992 and 2017. Manatees are commonly found in Buttonwood Sound, Florida Keys during the cooler months (November –May), but are less numerous in the warmer months (June –October). Sponges involved in other experiments (e.g., predation by starfish, competition, growth studies) were returned to Florida Bay by tie-wrapping the sponges to one or two cement bricks so that they would not die rolling around in the seagrasses. The species of sponge tied to bricks included those listed in Table 1. The bricks were placed a 1–2 m from the dock in about less than a meter of water, easily observed from the dock. They were monitored at least daily for approximately 1 week after placement.

**Table 1:** Consumption of sponges by the Florida manatee *T. manatus latirostris*.

Species of sponge	Consumed	Chemically defended?	Properties of spicules
*Chondrilla caribensis*^1^	YES	V	spherasters
*Spheciospongia vesparium*^1^	NO	V	monaxon
*Anthosigmella varians*^2^	NO	U	monaxon
*Clathria* sp. 1^1^ (*virgultosa?*)	NO	D	monaxon
*Ircinia felix*^1^	NO	D^a^	none (fiber meshwork)
*Lissodendoryx isodictyalis*^1^	NO	D	monaxon
*Haliclona magnifica*^1^	NO	D, V, U^a^	monaxon
*Halichondria melanodocia*^1^	NO	D	monaxon
*Tedania ignis*^1^	NO	V	monaxon
*Dysidea* sp.^1^ (*etheria?*)	NO	D	none (organic fibers)

**Notes.**

Chemically defended: U undefended V variable D defended

Properties of spicules: spherasters (round), monaxon (1 or 2-pointed).

Data from: 1 Loh & Pawlik, 2014 2 Hill & Hill, 2002

a other species in the genus.

A sample dock was selected in Buttonwood Sound in the Upper Keys (longitude 25.101908, lattitude −80.438824) after observing manatees eating *C. caribensis* from the pylons in the winter months. Evidence of *C. caribensis* predation by manatees included that the sponge (1) was shredded, (2) had missing layers, and (3) appeared white rather than the greyish brown color due to eating the outer sponge layer containing the symbiotic bacteria. Some of the *C. caribensis* were scraped completely off the pylons by manatees, while remnants of others were hanging from the pylons by thin strands of tissue. Such injuries of *C. caribensis* were monitored over a week-long period from December 4–8, 1995. We do not know the full recovery time in which the symbiotic cyanobacteria grows back and the color of the sponge returns to a greyish brown color, though it is longer than one week. Percent of eaten *C. caribensis* was estimated by point-intercept methods from all four sides of each of the 11 dock pylons holding up the dock. Clear plexiglass (ca. 0.33 × 0.33 m) was marked with dots 2.5 cm apart for a total of 121 dots. Each side of each pylon was scored from the surface of the water to the bottom as “eaten” or “uneaten” *C. caribensis* or “something else”. There were rarely other species of sponge found on the pylons. The sides of pylons were divided into two groups: those that were exposed to direct sunlight coming from the east, west or south (11 sides), and those that had no direct sunlight (33 sides).

Since manatees eat mostly seagrasses and algae, preference for *C. caribensis* that have more chlorophyll-*a* (from symbiotic cyanobacteria) in them was tested. A small piece of *C. caribensis* (1.75 cm^2^ wide circle by about 0.5 cm thick) was collected from each side of each pylon on December 8–9, 1995 and placed in a small vial. Chlorophyll-*a* per gram of wet weight and dry weight were determined from 8 pieces of *C. caribensis* on August 7, 1997. Four ml of acetone were added to each of these samples to extract the chlorophyll and the vial was placed in a freezer at −10 °C. After 24 hr the absorbance of the chlorophyll extract was read on a spectrophotometer, and the amount of chlorophyll-*a* (µg/cm^2^) was calculated (11.43*663 nm –0.64*630 nm) × (volume of the acetone).

The Florida Wildlife Commission sent stomach contents of a dead manatee (MNW17086) which was found in August 2017 near the Crystal River, Citrus County, Florida (longitude 28.922051, latitude −82.690457). It presumably died from exposure to red tide. While some areas have known resident populations, this manatee was not a known animal, so the Florida Wildlife Commission does not know its foraging range. The Florida manatee can range from Texas, to the Bahamas, and north to Massachusetts. The stomach sample was sent by mail from the Florida Wildlife Commission, and analyzed microscopically for the shape and size of spicules.

## Results

The Florida manatee was commonly observed over a twenty-five year period (1992–2017) removing and swallowing the sponge *C . caribensis* off pylons and hard surfaces both during the daytime and when it is dark in Buttonwood Sound, Florida Bay in the Florida Keys (Fig. 1). Most of the time the manatees were just swimming by, but when they stopped by a dock or seawall they appeared to be eating *C. caribensis.* The dark outside of the *C. caribensis* was shredded, exposing the white interior of the sponge, making the predation events very visible (Fig. 1). Unexpectedly it was found that at least 20 manatees (not identified except by size) over a 20 year period (1995–2014) made a choice of eating *C. caribensis* and no other sponges from the bricks during the week after they were put out (Supplementary data). There was no evidence of other predators on *C. caribensis*, and no such signs of manatee feeding on other species of sponge on the bricks, the bottom and sides of a dock. A small starfish *Echinaster spinulosus* is uncommonly found on *C. caribensis* in the mangroves of Florida Bay, but shows no evidence of removal of sponge tissue (probably a micro-predator), and moves off of *C. caribensis* in less than a day.

**Figure 2:**
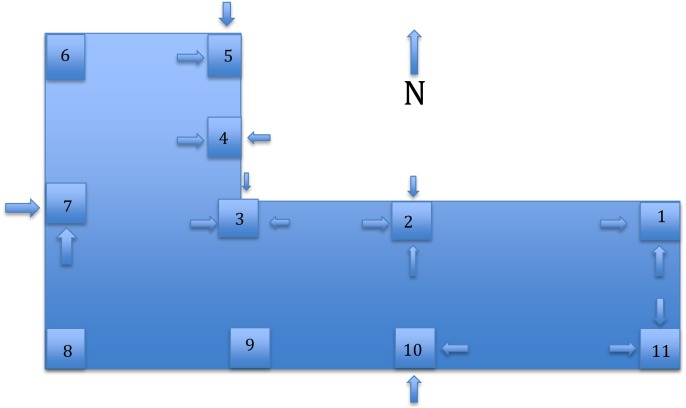
Pylons on a sample dock. A sample dock in Buttonwood Sound in Key Largo, Florida. The pylons are shown, with arrows denoting those sides from which manatees had eaten more that 10% of the *C. caribensis*. Dock was L-shaped, the longest side extending from the shore about 50’ and the short side in deeper water about 25’ long. Pylons were ca. 0.33 × 0.33 m square, about 5 m apart, in 1–2 m of water, and completely covered with benthic invertebrates and algae.

**Figure 3:**
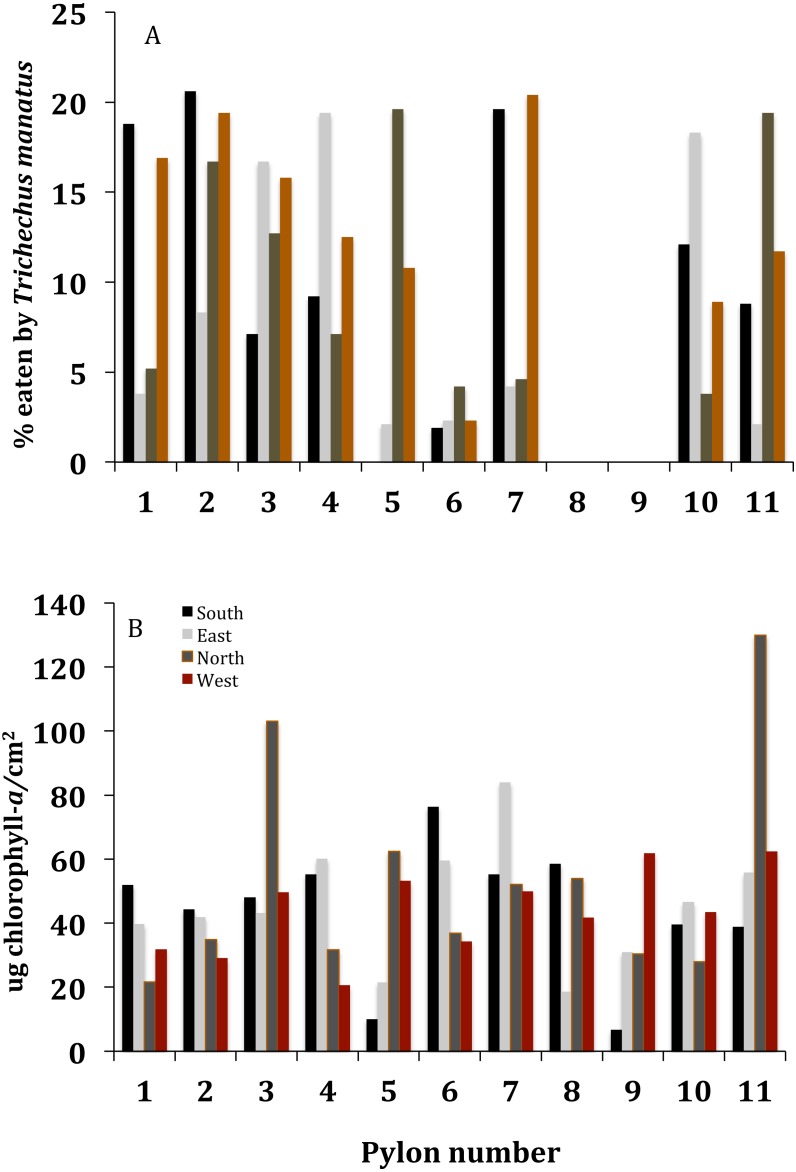
The percent of *C. caribensis* eaten off pylons of sample dock, along with the amount of chlorophyll-a in the sponge. (A) The percent of *C. caribensis* eaten off each side of the eleven pylons of the sample dock. (B) The µg chlorophyll-*a* per cm^2^ of a representative piece of *C. caribensis* from each side of the eleven pylons of the sample dock.

Manatees fed mostly on the interior and north sides of pylons of the sample dock (Fig. 2). Manatees ate an average of 10.8 ± 6.8% (s.d., *n* = 44) of the *C. caribensis* off the sides of pylons (Figs. 1, 3A), where the percent cover varied from approximately 5 to about 50%. There were 15/33 (45.5%) sides of pylons with no direct sunlight that had >10% *C. caribensis* eaten and only 3/11 (27.3%) sides of pylons that were facing either east, west or south with direct sunlight that had at least 10% eaten (Figs. 2, 3A). The value of >10% was picked because the average that the manatees ate was close to this (10.8 ± 6.8%). The two pylons that showed no signs of grazing by manatees had anemones (*Aiptasia pallida*) occurring on the *C. caribensis,* whereas the other sides of pylons had no visible anemones on the sponge.

The µg chlorophyll-*a*/cm^2^ of *C. caribensis* tissue is relatively constant on all sides of the pylons, with an average value of 46.6 ± 22.6 (s.d., *n* = 44) (Fig. 3B). Mean values of 19.3 ± 12.1 µg chlorophyll-a/g wet weight or 107.9 ± 64.7 chlorophyll-a/g dry weight were recorded (95% CI, *n* = 8). The percent of *C. caribensis* eaten as related to µg chlorophyll-*a/* cm^2^ is not significant (linear regression *r*^2^ = 0.04, *p* > 0.05, *n* = 44).

**Figure 4:**
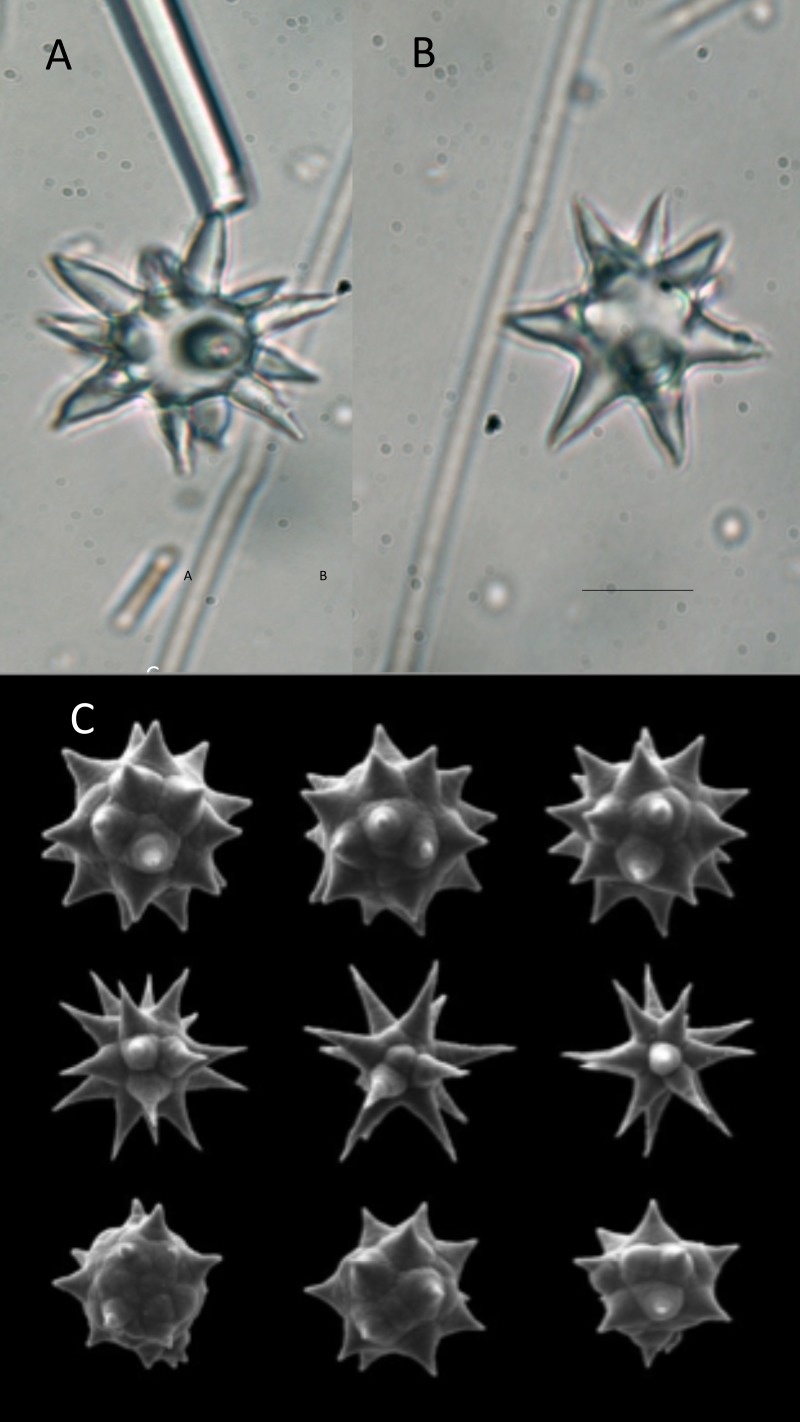
Spicules found in dead manatee MNW17086. (A, B) Microscleres of Chondrilla (caribensis?) from the stomach of manatee MNW 17086 (light background); (C) Microscleres from type specimens of C. caribensis (dark background, Rützler, Duran & Piantoni, 2007). Dark bar is 10 µm long.

Spicules from a dead manatee (FSW17086) were analyzed, and found to contain more than one species of sponge (e.g., several sharp monaxon and tetraxon vs. round spheraster spicules). There were many *Chondrilla*-type spheraster microscleres seen in the sample (Fig. 4), but it was difficult to tell if any of the species of sponge was directly or incidentally eaten.

## Discussion

H1: Manatees “select” the sponge *C. caribensis* from others.

Manatees in Florida are known to eat barnacles, bivalves, gastropods, crabs, and tunicates (O’Shea et al., 1991; Courbis & Worthy, 2003). Powell (1978) reported carnivory by Antillean manatees of fish from gill nets in Jamaica. While it is unusual for any marine animal to eat chemically defended prey, such as certain sponge species (Green, 1977; Swearingen & Pawlik, 1998), the Florida manatees select and consume the sponge *C. caribensis*, removing *C. caribensis* off of docks, pylons, seawalls, or any hard surface. There was no evidence that any other species of sponge was directly consumed by manatees, though this may be possible with some sponges containing variable or no chemical defenses and/or round vs sharp spicules. Sponges from other experiments that were not eaten by manatees were typically chemically defended or had sharp monaxon spicules (e.g., Table 1, Jones, Blum & Pawlik, 2005).

H2: Manatees eat varying amounts (>10%) of *C. caribensis*

Manatees ate over half of the average cover eaten of *C. caribensis* (over 10%) from sides of pylons of the sample dock in Florida Bay (Figs. 1–3). Six docks near the sample dock, many pylons, seawalls, and hard surfaces show signs of manatees feeding on *C. caribensis.*

H3: Manatees prefer eating *C. caribensis* in the shadiest area of the sample dock

Manatees in this study are about twice as likely to forage in the shady area vs. a sunny portion of the dock. The shadier areas were usually sides of pylon surfaces that were completely covered by the dock, compared to the sides of pylons that were exposed for some time to direct sun (the eastern, southern, and western sides that were not fully shaded by the dock). This could be due to desire of manatees to minimize exposure to ultra-violet light, maximize cooler conditions, avoid boats and/or humans, or it could be some unknown aspect of consumption of *C. caribensis* by manatees.

H4: Manatees eat *C. caribensis* containing more chlorophyll

It was thought that because manatees forage seagrasses and algae, they would also prefer eating a sponge containing high amounts of chlorophyll, possibly for nutritional purposes. However, manatees apparently do not distinguish *C. caribensis* based on the amount of chlorophyll-*a* (*p* > 0.05). There is no evidence that any aquatic mammal can detect the relative amount of chlorophyll in food.

Why do manatees consume *C. caribensis*? Manatees and dugongs incidentally consume invertebrates that occur on the blades of seagrasses (e.g., O’Shea et al., 1991; Allen et al., 2017), which would include sponges, however this is not considered active feeding on sponges. While manatees may be eating *C. caribensis* because local food resources might be diminished, possibly due to global warming (e.g., O’Shea, 1986), some scientists propose that manatees eat invertebrates for the nitrogen within. Manatees living in marine habitats eat mostly seagrasses, which take-up nitrogen mostly through their leaves and are thought to be nitrogen limited (Duarte, 1990; Nayer et al., 2018). Up to 50% of nitrogen requirements of seagrasses are supplied by epiphytes living on the leaves, and seagrasses provide 8–22% protein per dry weight equivalent to corn and wheat (Walsh & Grow, 1972). It certainly seems possible that manatees could supplement their nitrogen (protein) consumption by eating sponges.

While protective compounds are normally found in sponges, those are evidently not as potent in *C. caribensis* as in other sponges (Table 1), enabling predation by fish and turtles (Randall & Hartman, 1968; Chanas & Pawlik, 1995; Swearingen & Pawlik, 1998; Pawlik, Loh & McMurray, 2018). The “mechanical defenses” include siliceous spherasters (round) in *C. caribensis*, rather than pointed spicules as in other species. *C. caribensis* has minute secondary spines on round spicules (Rützler, Duran & Piantoni, 2007) that apparently interfere minimally with consumption by turtles (Meylan, 1988; Bjorndal, 1990; Pawlik, Loh & McMurray, 2018), fish and manatees. It appears that *C. caribensis* has a combination of variable chemical defenses coupled with round rather than pointed spicules (Table 1), that enables manatees to eat it.

## Conclusion

The manatee *T. manatus latirostris* has been observed to directly feed on the sponge *C. caribensis*. This sponge has a symbiotic relationship with cyanobacteria, but the correlation of chlorophyll-*a* with percent consumed is insignificant. Manatees eat more of *C. caribensis* from the shaded areas under a dock compared to non-shaded areas, which could be a defense of manatees to boats, humans, or both. *C. caribensis* has variable chemical defenses along with round (rather than pointed) spicules, which could explain its palatability to manatees.

##  Supplemental Information

10.7717/peerj.8443/supp-1 Supplemental Information 1 Data for Figures 3 and 4

10.7717/peerj.8443/supp-2 Supplemental Information 2 Manatees seen eating C. caribensis off bricks

## Additional Information and Declarations

### Data Availability

The following information was supplied regarding data availability: Raw data are available as a Supplemental File.
