# Supplementary material for: Florida manatees Trichechus manatus latirostris actively consume the sponge Chondrilla caribensis
Source: PeerJ. 2020 Jan 22;8:e8443. doi: 10.7717/peerj.8443 (PMC6982412; doi:10.7717/peerj.8443)
Supplement: Supplemental Information 2 [file peerj-08-8443-s002.docx]

**Table.** Time of year cement bricks with sponges were placed by the dock, with number of manatees seen eating *C. caribensis* from the bricks sometime during the following week.

Time of year Number of manatees

December 1995 1

June 1996 3

July 1996 7

March 1997 1

March 1998 2

March 2000 1

March 2002 2

March 2012 1

April 2013 1

June 2014 1
